# MiRNA and associated inflammatory changes from baseline to hypoglycemia in type 2 diabetes

**DOI:** 10.3389/fendo.2022.917041

**Published:** 2022-08-09

**Authors:** Manjunath Ramanjaneya, Ruth Priyanka, Milin Bensila, Jayakumar Jerobin, Krunal Pawar, Thozhukat Sathyapalan, Abdul Badi Abou-Samra, Najeeb M. Halabi, Abu Saleh Md Moin, Stephen L. Atkin, Alexandra E. Butler

**Affiliations:** ^1^ Qatar Metabolic Institute, Hamad Medical Corporation, Doha, Qatar; ^2^ Translational Research Institute, Hamad Medical Corporation, Doha, Qatar; ^3^ Amity Institute of Biotechnology, Amity University, Jaipur, India; ^4^ Academic Endocrinology, Diabetes and Metabolism, Hull York Medical School, Hull, United Kingdom; ^5^ Weill Cornell Medicine in Qatar, Education City, Qatar Foundation, Doha, Qatar; ^6^ Research Department, Royal College of Surgeons in Ireland Bahrain, Adliya, Bahrain

**Keywords:** Type 2 diabetes, hypoglycemia, miRNA, metabolic pathways, proteomics

## Abstract

**Objective:**

Hypoglycemia in type 2 diabetes (T2D) increases morbidity and mortality but the underlying physiological response is still not fully understood, though physiological changes are still apparent 24 hours after the event. Small noncoding microRNA (miRNA) have multiple downstream biological effects that may respond rapidly to stress. We hypothesized that hypoglycemia would induce rapid miRNA changes; therefore, this pilot exploratory study was undertaken.

**Methods:**

A pilot prospective, parallel study in T2D (n=23) and controls (n=23). Insulin-induced hypoglycemia (2mmol/l: 36mg/dl) was induced and blood sampling performed at baseline and hypoglycemia. Initial profiling of miRNA was undertaken on pooled samples identified 96 miRNA that were differentially regulated, followed by validation on a custom designed 112 miRNA panel.

**Results:**

Nine miRNAs differed from baseline to hypoglycemia in control subjects; eight were upregulated: miR-1303, miR-let-7e-5p, miR-1267, miR-30a-5p, miR-571, miR-661, miR-770-5p, miR-892b and one was downregulated: miR-652-3p. None of the miRNAs differed from baseline in T2D subjects.

**Conclusion:**

A rapid miRNA response reflecting protective pathways was seen in control subjects that appeared to be lost in T2D, suggesting that mitigating responses to hypoglycemia with blunting of the counter-regulatory response in T2D occurs even in patients with short duration of disease.

**Clinical trial registration:**

https://clinicaltrials.gov/ct2/show/NCT03102801?term=NCT03102801&draw=2&rank=1, identifier NCT03102801.

## Introduction

Severe hypoglycemia in type 2 diabetes (T2D) is associated with cardiovascular-related events that are a risk for increased mortality (1–6), observations that have been reflected in the reports from both retrospective and longitudinal cohort studies (7–10). Causative factors underlying the clinical observations resulting from hypoglycemia include endothelial and thrombotic dysfunction (11–13), oxidative and inflammatory stress (14, 15) and the heat shock protein response (16), but the diverse range of underlying mechanistic dysfunctions resulting from hypoglycemia have not been completely clarified.

MicroRNAs (miRNAs) are non-coding RNAs of ~22 nucleotides in length that function post-transcriptionally as regulators of gene expression; their inhibitory actions lead to mRNA cleavage, repression of translation and mRNA decay (17–20). Each miRNA may impact expression of multiple target mRNAs, thus affecting an array of cellular and biologic pathways. MiRNAs are thought to play a role in many disease states including diabetes and its associated co-morbidities (21, 22). A growing body of evidence shows the critical role of miRNAs in cellular stress responses (23), such as in diabetes, where, for example, miR-126 is involved in vascular repair (24), miR-146 is associated with proinflammatory factors and oxidative stress (25) and knockdown of miR-185 may increase oxidative stress (26, 27). What is also apparent is that the miRNA response can be relatively rapid and may occur within one hour of a stress event (23, 28, 29).

Changes in glucose may modulate miRNA changes with diminished expression of miR-215-5p miR-296-5p and miR-497-3p levels in response to high glucose in the human myometrium (30), and miRNA-375 (miR-375), miRNA-155 (miR-155), miRNA- 21 (miR-21), miRNA-33 (miR-33), the let-7 family have been highlighted to be related to and glucometabolic regulation (31).

*In vitro*, the low glucose condition decreased the expression of miRNA-17-5p and miRNA-20a-5p in hepatoma cells (32), whilst several miRNA were modulated by low glucose in embryonal mouse hypothalamus cells (33). In animal models, hypoglycemia resulted in ventromedial hypothalamus downregulation of miR7a-5p that, when targeted overexpression was used, the epinephrine response to hypoglycemia was restored (34). In a study restricted to 14 patients with type 2 diabetes alone who underwent a hypoglycemic challenge, selective miRNA analysis restricted to those associated with platelet expression was undertaken on hsa-miR-106a.5p, hsa-miR-126-3p, hsa-miR-126-5p, hsa-miR-15a-5p, hsa-miR-15b-3p, has-miR-15b-5p, hsa-miR-16-5p, hsa-miR-223-3p, hsa-miR-223-5p, hsa-miR-129-2-3p. The results focused on longer term changes of 1 day and 7 days after the hypoglycemic episode but showed that miR-106a-5p, miR-15b, miR-15a, miR-16-5p, miR-223 and miR-126 were increased (35). However, the miRNA response and the rapidity of the response to the development of hypoglycemia in humans remains unknown, but hypothetically the rapid modulation of miRNA may drive subsequent pathophysiological changes; therefore, this pilot exploratory study was undertaken.

## Methods

### Study design

This pilot prospective parallel study was performed in 46 Caucasian subjects (age range 40–70 years), T2D (n = 23) and control (n = 23), at the Diabetes Centre at Hull Royal Infirmary (36). All T2D patients had diabetes duration <10 years and treatment with a stable dose of medication (metformin, angiotensin converting enzyme inhibitor/angiotensin receptor blocker and/or statin) over the 3-month period prior to participation in the study. For anti-diabetic therapy in the T2D group, only metformin was allowed and HbA1c was required to be <10% (86 mmol/mol), with no hypoglycemic unawareness/hypoglycemia within the 3-month period prior to participation, and no diabetes related complications. For control subjects, an oral glucose tolerance test (OGTT) was used to exclude diabetes. All participants had body mass index (BMI) of 18-49kg/m^2^, normal screening blood biochemistry, no cancer history or contraindication to undertake insulin infusion to hypoglycemia.

### Study participants

A medical history with clinical examination, routine blood tests plus an electrocardiogram was performed on all participants. Hypoglycemia was induced by a continuous insulin infusion, as previously detailed (11), with blood samples taken at baseline and hypoglycemia. The infusion from baseline to severe hypoglycemia was over a 1-hour timeframe with blood glucose dropping from 3.5 to 2 mmol/l over an approximately 25-minute period.

Each participant provided written and signed informed consent, and the trial conducted per the Declaration of Helsinki. Approval for the trial was granted by the North West-Greater Manchester East Research Ethics Committee (REC number:16/NW/0518); trial registration at www.clinicaltrials.gov (NCT03102801) on 06/04/2017.

### Insulin infusion

The insulin infusion was performed as previously detailed (11). “Following an overnight fast, 30–60 min prior to the commencement of the test (0830 h), bilateral ante-cubital fossa indwelling cannulas were inserted. To induce hypoglycemia, soluble intravenous insulin (Humulin S, Lilly, UK) was given in a pump starting at a dose of 2.5 mU/kg body weight/min with an increment of 2.5 mU/kg body weight/min every 15 min by hypoglycemic clamp, until two readings of capillary blood glucose measured by a glucose analyser (HemoCue glucose 201 +) ≤ 2.2 mmol/L (< 40 mg/dl) or a reading of ≤ 2.0 mmol/L (36 mg/dl) was achieved. Initially, patients with T2D were clamped to euglycemia (5mmol/l; 90mg/dl), then subsequently to hypoglycemia. The blood sample schedule was timed subsequently in respect to the time point that hypoglycemia occurred. Following the identification of hypoglycemia, intravenous glucose was given in the form of 150 ml of 10% dextrose and a repeat blood glucose check was performed after 5 min if blood glucose was still < 4.0 mmol/L”.

### Biochemical markers

As previously described (36), “blood samples were separated immediately by centrifugation at 2000 g for 15 min at 4°C, and the aliquots were stored at -80°C, within 30-min of blood collection, until batch analysis. Fasting plasma glucose (FPG), total cholesterol, triglycerides, and high-density lipoprotein (HDL) cholesterol levels were measured enzymatically using a Beckman AU 5800 analyser (Beckman-Coulter, High Wycombe, UK)”.

### RNA extraction from human plasma

Total RNA was extracted using the MagMAX™ mirVana™ RNA Isolation Kit (Thermo Fischer Scientific, Waltham, MA, USA) on an automated KingFisher instrument (Thermo Fischer Scientific, Waltham, MA, USA). The MagMAX™ mirVana™ RNA isolation kit is a magnetic bead-based kit that uses MagMAX magnetic-bead technology for efficient isolation of total RNA from plasma samples from 100 µl of human plasma.

### Expression analysis of microRNA by taqman openarray human advanced microRNA panel

2 µl of the extracted total RNA was used for cDNA conversion using TaqMan™ Advanced miRNA cDNA Synthesis Kit as per manufacturer’s recommended protocol. Given the low abundance of miRNA in plasma samples, cDNA was preamplified prior to the final real-time PCR step. 1:20 diluted cDNA was mixed with 2X TaqMan OpenArray Real-Time Master Mix solution to perform real-time PCR on the OpenArray plate. 5 µL sample of PCR reactions was pipetted into each well of a 384-well plate and samples with the master mix were loaded from the 384-well sample plate onto the OpenArray plate using the OpenArrayAccuFill System. The PCR was performed on the QuantStudio 12K Flex Real-Time PCR System (Thermo Fischer Scientific, Waltham, MA, USA). The miRNA measurements were done in two phases. 1) profiling and 2) validation. Initially profiling was done using pooled samples (4 in each group) obtained from 12 subjects in both control and diabetes group at baseline and at hypoglycemia. Profiling for miRNA was performed using standard The TaqMan^®^ OpenArray^®^ Human MicroRNA Panel, QuantStudio™ 12K Flex panel which is a fixed content panel that contains 754 well-characterized human miRNA sequences. Profiling was done to identify lead targets for validation according to the manufacturer’s recommended protocol. Profiling experiments were done in a set of pooled samples taken from both the baseline and hypoglycemia timepoints and using default miRNA panels which consisted of 754 miRNAs. From this pooled sample data analysis, 96 miRNA that were differentially regulated were selected for the validation phase. Validation was done on custom designed 112 miRNA panel which included 96 miRNAs identified from the profiling phase, internal controls and other miRNAs that were published in the literature to be differentially expressed in patients with diabetes-related complications. The miRNA validation was done on diabetic and control subject samples, n=23 per group, at baseline and at hypoglycemia. The miRNA expressions were quantified using ExpressionSuite Software (Thermo Fischer Scientific, Waltham, MA, USA) data-analysis tool that utilizes the comparative Cτ (ΔΔCτ) method to accurately quantify relative miRNA expressions. ExpressionSuite generates the results in the form of Rq values, which are equivalent to the log2 scale, therefore any changes indicate a log2 fold change.

Ingenuity Pathway Analysis (IPA) software (Qiagen, Germantown, Maryland, USA) allows for data analysis together with integration of data derived from an array of experimental datasets, including gene expression and miRNA. Here, we performed IPA to illustrate the canonical pathways related to the top 10 altered miRNAs in control and T2D subjects highlighted in this study.

### Statistics

There was no information regarding changes in miRNA on which to base a sample size calculation. For such pilot studies, Birkett and Day (37) suggest a minimum of 20 degrees of freedom to estimate variance from which a larger trial could be powered, hence 23 subjects in each group were recruited. SPSS (v22, Chicago, Illinois) was used for statistical analysis. Descriptive data is presented as mean ± SD for continuous data and n (%) for categorical data. T-tests or Mann Whitney tests were used to compare means/medians as appropriate. Mean normalization was performed before analysis using the global mean of each miRNA. Normalization was performed using GenEx software (provided with the Bioanalyzer) to achieve a global mean of all miRNAs with Ct <35. An unpaired t-test was used to test paired changes between T2D and controls in miRNA levels.

## Results

Demographic and clinical characteristics of the study participants are shown in **Table 1**. Those subjects with T2D were of relatively short 4.5-year disease duration (38), were free of microvascular and macrovascular complications, had an increased BMI, and with lower total cholesterol and HDL compared to controls.

**Table 1:** Demographic and clinical characteristics of the study participants.

Baseline	Type 2 Diabetes (n=23)	Controls (n=23)	p-value
Age (years)	64 ± 8	60 ± 10	0.15
Sex (M/F)	12/11	11/12	0.77
Weight (kg)	90.9 ± 11.1	79.5 ± 8.8	<0.0001
Height (cm)	167 ± 14	169 ± 5	0.64
BMI (kg/m^2^)	32 ± 4	28 ± 3	<0.0001
Duration of diabetes (years)	4.5 ± 2.2	N/A	
HbA1c (mmol/mol)	51.2 ± 11.4	37.2 ± 2.2	<0.0001
HbA1c (%)	6.8 ± 1.0	5.6 ± 0.2	<0.0001
Total cholesterol (mmol/l)	4.2 ± 1.0	4.8 ± 0.8	0.014
Triglyceride (mmol/l)	1.7 ± 0.7	1.3 ± 0.6	0.06
HDL-cholesterol (mmol/l)	1.1 ± 0.3	1.5 ± 0.4	0.001
LDL-cholesterol (mmol/l)	2.2 ± 0.8	2.7 ± 0.9	0.05
CRP (mg/l)	3.1 ± 2.9	5.3 ± 0.3	0.66

BMI, Body mass index; BP, Blood pressure; HDL-cholesterol, High density lipoprotein cholesterol; LDL-cholesterol, Low density lipoprotein cholesterol; CRP, C-reactive protein; HbA1c, Hemoglobin A1c.

All subjects experienced neuroglycopenic symptoms, feeling tremulous and sweating, as blood glucose was lowered, though this was transient and reversed quickly as soon as the hypoglycemic target was reached which was immediately reversed with dextrose.

**Figure 1:**
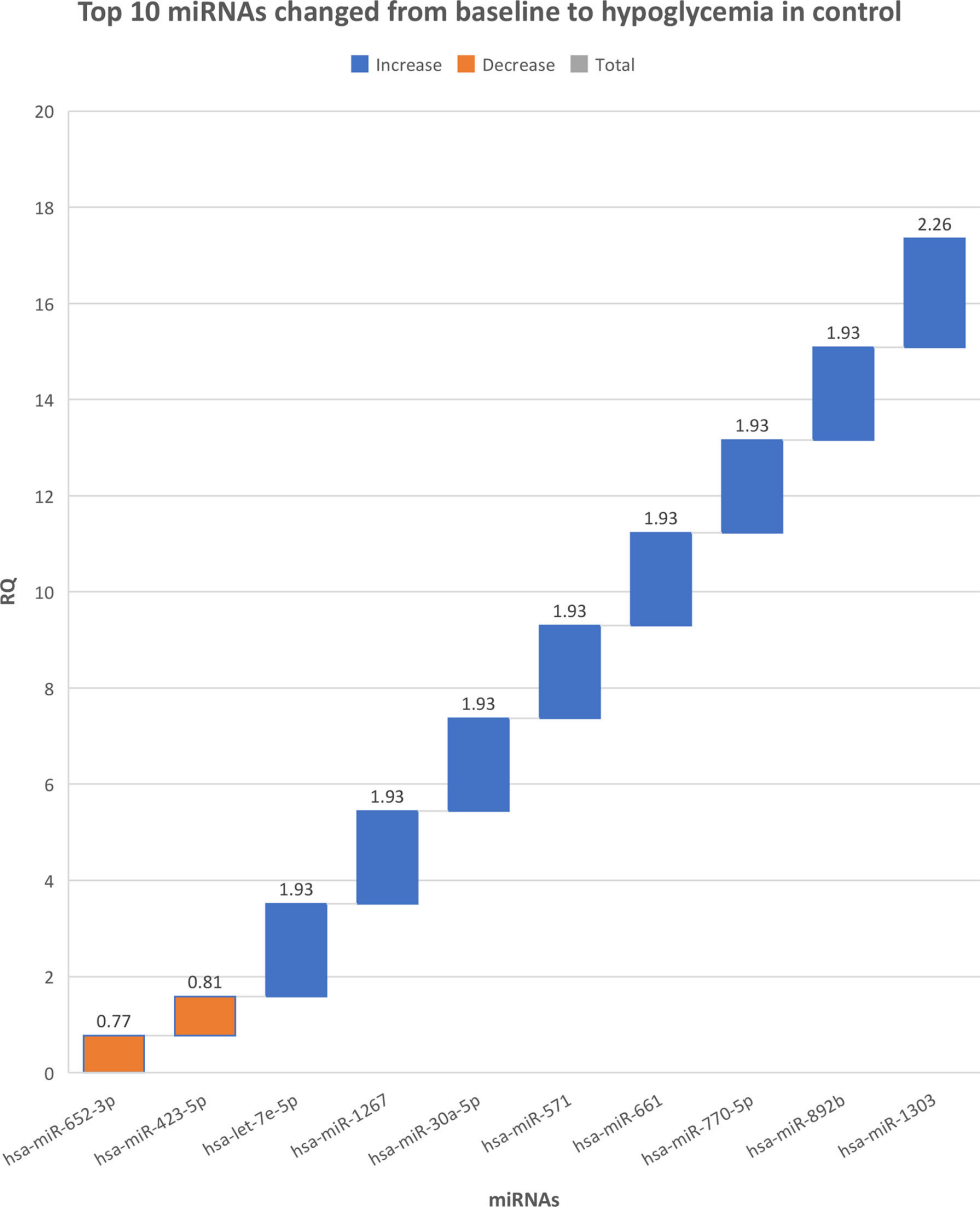
The top 10 miRNAs that changed from baseline to hypoglycemia in control subjects. RQ, mean relative quantification.

**Figure 2:**
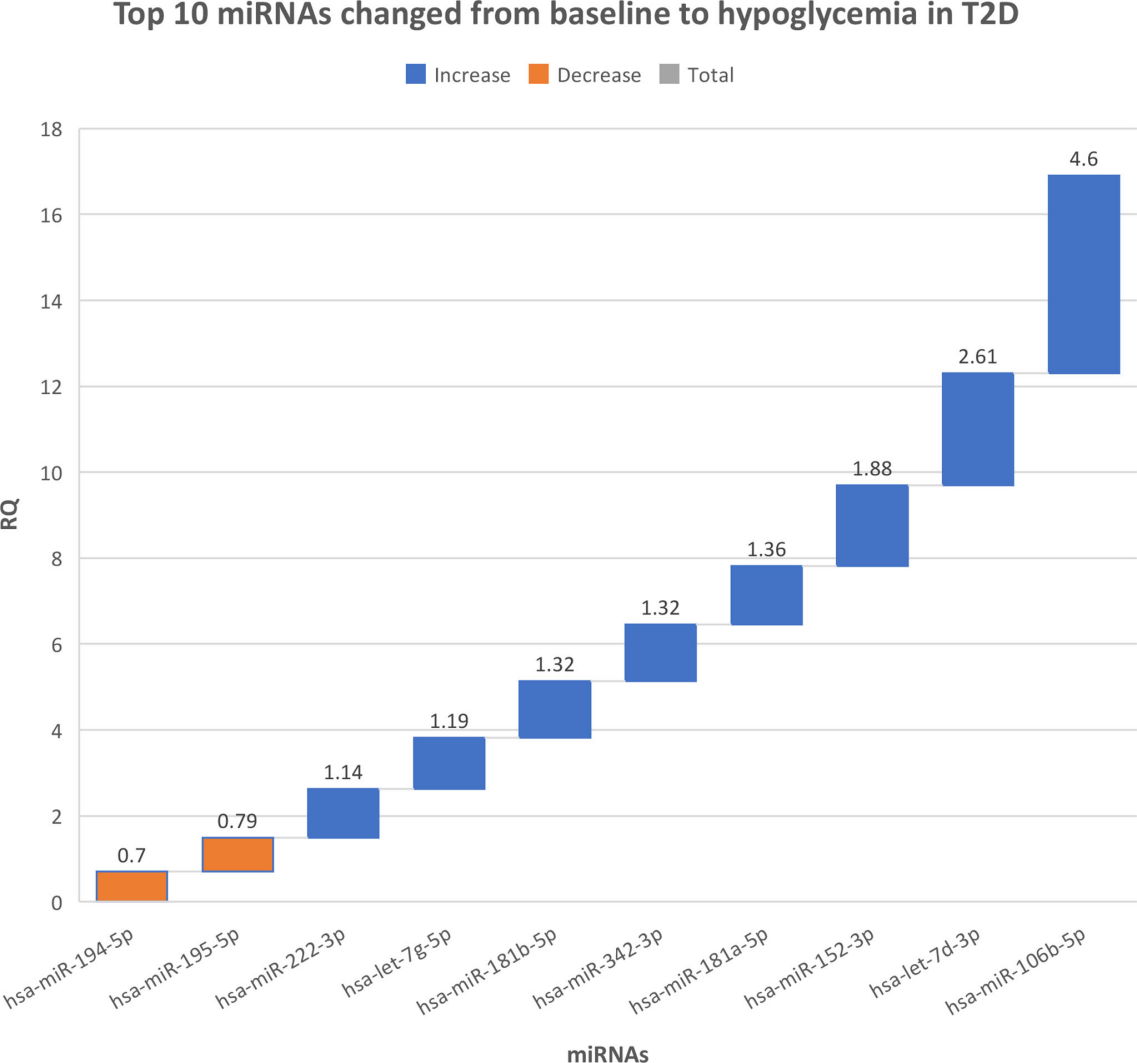
The top 10 miRNAs that changed from baseline to hypoglycemia in type 2 diabetes subjects. RQ, mean relative quantification.

There were no differences in miRNAs between T2D and controls at baseline, as shown in **Supplementary Table 1**, or at hypoglycemia. The top 10 miRNA with the greatest changes from baseline to hypoglycemia are shown in **Table 2**, in which it can be seen that 9 of the miRNAs differed significantly in controls (eight were upregulated: miR-1303, miR-let-7e-5p, miR-1267, miR-30a-5p, miR-571, miR-661, miR-770-5p and miR-892b, whilst one was downregulated: miR-652-3p); mean Relative Quantification (RQ) fold changes for these top 10 miRNAs are shown in **Figure 1**. Notably, no miRNAs differed significantly from baseline to hypoglycemia in T2D subjects, though mean RQ fold changes for the top 10 miRNAs that showed differing expression are shown in **Figure 2**.

**Table 2:** Top 10 miRNAs that changed from baseline to hypoglycemia in T2D and control subjects in the validation experiment.

CONTROLS			T2D		
Target Name	Rq	P-Value	Target Name	Rq	P-Value
hsa-miR-1303_478698_mir	2.26	0.01	hsa-miR-106b-5p_478412_mir	4.60	0.06
hsa-miR-652-3p_478189_mir	0.77	0.02	hsa-miR-194-5p_477956_mir	0.70	0.11
hsa-let-7e-5p_478579_mir	1.93	0.03	hsa-let-7g-5p_478580_mir	1.19	0.14
hsa-miR-1267_478672_mir	1.93	0.03	hsa-let-7d-3p_477848_mir	2.61	0.15
hsa-miR-30a-5p_479448_mir	1.93	0.03	hsa-miR-181b-5p_478583_mir	1.32	0.17
hsa-miR-571_479054_mir	1.93	0.03	hsa-miR-152-3p_477921_mir	1.88	0.17
hsa-miR-661_479144_mir	1.93	0.03	hsa-miR-195-5p_477957_mir	0.79	0.17
hsa-miR-770-5p_479178_mir	1.93	0.03	hsa-miR-181a-5p_477857_mir	1.36	0.18
hsa-miR-892b_479198_mir	1.93	0.03	hsa-miR-222-3p_477982_mir	1.14	0.19
hsa-miR-423-5p_478090_mir	0.81	0.07	hsa-miR-342-3p_478043_mir	1.32	0.21

Rq, relative level of miRNA expression.

All of the 112 miRNA that were measured in this customized panel in the validation phase of this study are shown in **Supplementary Table 2**.

**Figure 3:**
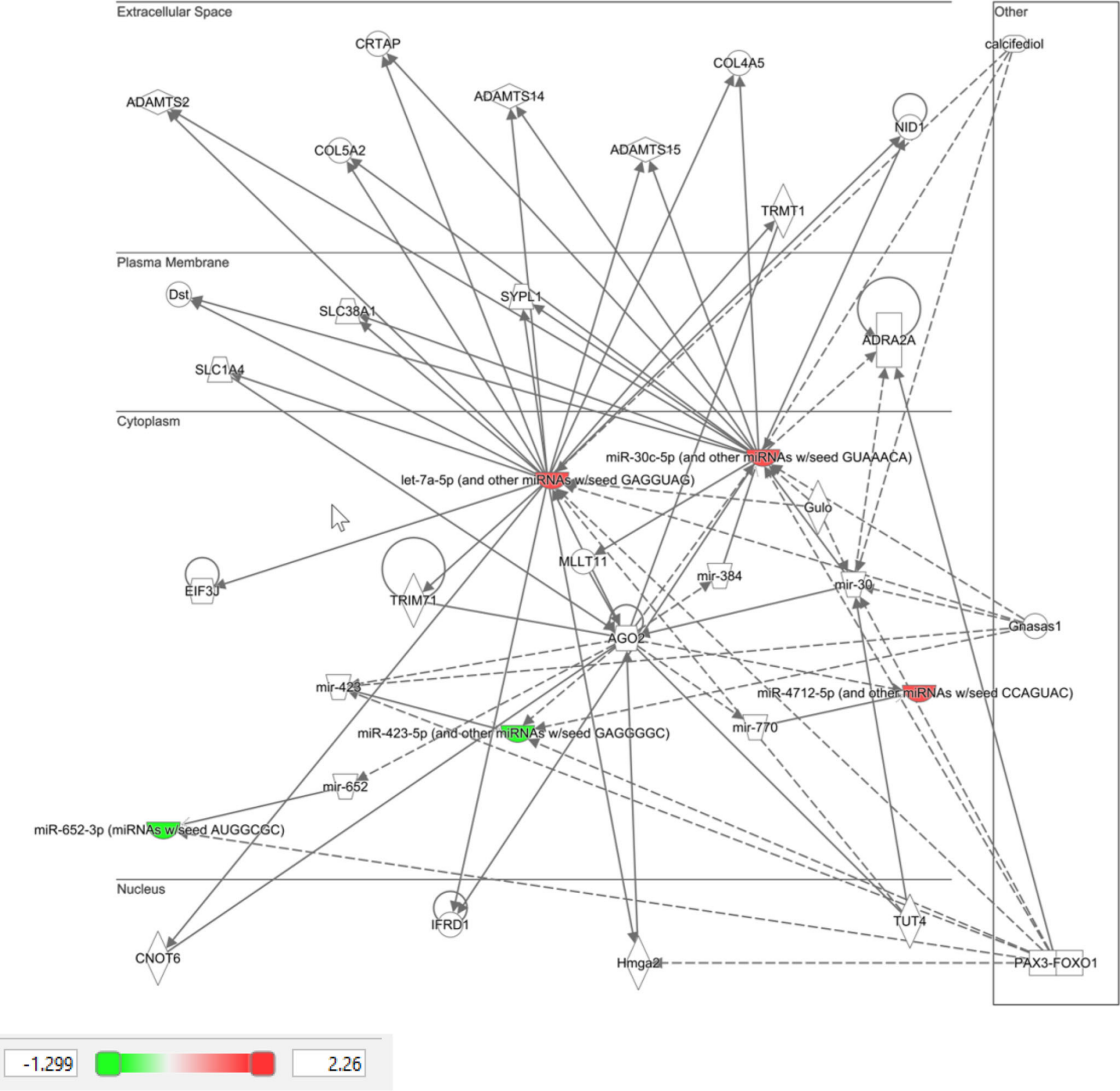
Ingenuity pathway analysis of top 10 miRNA that showed differential expression in control subjects in response to hypoglycemia.

**Figure 4:**
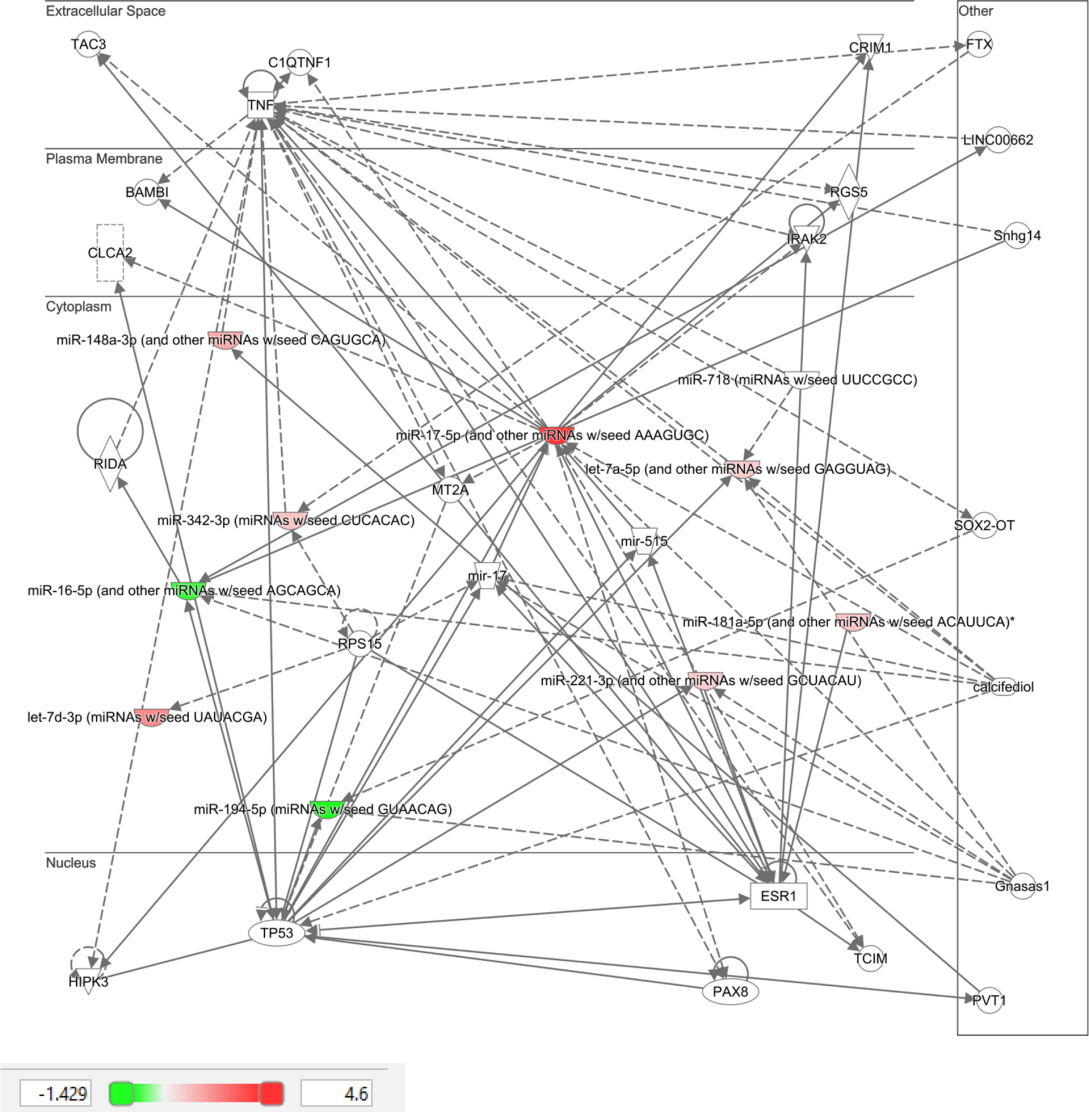
Ingenuity pathway analysis of top 10 miRNA that showed differential expression in type 2 diabetic subjects in response to hypoglycemia.

Ingenuity Pathway Analysis (IPA) was performed on the top 10 altered miRNAs in control (**Figure 3**) and T2D (**Figure 4**) subjects. Specifically focusing on the top 10 miRNAs found in T2D (as shown in **Table 2**), nine of these miRNAs were associated with neurological disease giving a score of p<0.04–p<4^14^ (the score calculates the likelihood that the network eligible molecules are found as part of the network by random chance alone; mathematically the score is the negative exponent of the right-tailed Fisher’s exact test). In addition, nine of these miRNAs were associated with organismal injury and abnormalities that are associated with diabetes complications, with a score of p<0.05–p<4^14^.

## Discussion

The novelty of this study was the demonstration that miRNAs are both up-regulated and down-regulated at hypoglycemia, an insult that occurred within 1-hour of initiating the insulin infusion, and that these changes were only seen in controls and not in T2D. There have been no human studies in the literature detailing early miRNAs changes in response to modulation of blood glucose to hypoglycemia with which to compare, and therefore future studies will be needed to confirm these findings. In a study restricted to 14 patients with type 2 diabetes who underwent a hypoglycemic challenge but without a control population, selective miRNA analysis derived from platelet expression showed that upregulation of the miRNA was seen at 1 day and 7 days but no miRNA analysis was reported in the first 24 hours (35). The control population in this study were diabetes free and represented an optimal response to hypoglycemia and showed that the miRNA changes seen were marked and differed significantly from the T2D subjects, suggesting that even in patients with a short duration of diabetes (i.e. less than 10 years after which hypoglycemic unawareness may develop, the physiological response to hypoglycemia is altered and perhaps blunted, a phenomenon better recognised in patients with T2D of longer duration (39, 40). In animal models, experimentally induced recurrent hypoglycemia resulted in ventromedial hypothalamus downregulation of miR7a-5p that, when targeted overexpression was used, the epinephrine response to hypoglycemia was restored (34). Overall, many of the miRNAs that were significantly different in controls have been associated with potential beneficial actions, suggesting that their changes may be protective, and that this protection is lost, or potentially delayed, in T2D. Previously, we have looked at metadrenaline and nor-metadrenaline in a hypoglycemic study in a separate cohort that showed differences at 24-hours but not at the point of hypoglycemia (15) and therefore these were not measured in this study. It is well described in the literature that the growth hormone and cortisol responses occur following the onset of hypoglycemia and, as the samples for miRNA measurement were taken at the exact timepoint of hypoglycemia, they would not be expected to be elevated as indicated by prolactin levels that did not differ either between or within groups (data not shown).

IPA clearly showed that the top 10 miRNAs in T2D are likely to be part of specific disease pathways; the neurological disease pathway encompasses Alzheimer’s disease that is associated with type 2 diabetes (41) and the organismal injury and abnormalities pathway that is associated with diabetes complications. While no individual miRNA was significant in T2D due to patient variability, the top 10 most significant miRNAs were significantly associated with specific diseases that may be diabetes related.

MiR-1303 was upregulated by hypoglycemia and has been associated with T2D, particularly in patients with complications (42); however, its exact role in diabetes is unclear. Within tumor biology, miR-1303 is regulated by the long non coding RNA BCRT1, and the increase in miR-1303 was associated with decreased cellular proliferation that may be of importance in microvascular complication development and therefore may be protective in this case.

MiR-let-7e-5p was also upregulated by hypoglycemia and has been shown to be upregulated by exercise (43), suggesting that stimulus-driven higher levels may be of benefit. MiR-let-7e-5p is down-regulated in DVT patients and overexpression of miR-let-7e-5p enhances the ability for thrombus revascularization in a rat model of venous thrombosis (44), suggesting a beneficial effect on thrombosis; however, its role in diabetes is unclear at present.

MiR-1267 was upregulated by low glucose and has been noted to be upregulated in podocytes (45), suggestive of a possible role in diabetic nephropathy, though this is speculative.

MiR-30a-5p was upregulated and has been reported to be a potential biomarker for T2D (46). In tumor biology and other models, upregulation is associated with a reduction of cellular growth (47) and a reduction of oxidative stress (48), and its upregulation may be nephroprotective against high glucose (49).

MiR-571 was upregulated by hypoglycemia and, together with miR-1303, it has been associated with T2D, particularly in patients with complications (42), most particularly microvascular complications (50). Similarly, miR-661 was upregulated and, together with miR-1303 and miR-571, it has been related to the development of microvascular complications (50), including nephropathy (42).

MiR-770-5p was upregulated in low glucose and its expression has been associated with podocyte inflammation and apoptosis (51, 52) and, together with miR-661 and miR-571, it was associated with diabetes microvascular complications (50).

MiR-892 was upregulated and has been shown to activate NF-kB, with a reduction in cellular proliferation in tumour models (53), and may have an effect on angiogenesis but its role in the development or protection against diabetes related complications needs clarification.

MiR-652-3p was downregulated by hypoglycemia, and, in a meta-analysis, its reduction was associated with T2D (54); its downregulation by statins has a protective effect on the endothelium (55) and perhaps its downregulation here is a reflection of a protective effect.

A study strength is that the T2D participants had short disease duration and relative treatment naïvete. Limitations include small study numbers, as a larger population may have shown additional miRNA changes. Additionally, the time course was limited to baseline to the point of hypoglycemia, though marked changes in miRNA levels in controls were apparent during that timeframe. The increased BMI of the T2D participants should not have altered the hypoglycemia-induced miRNA changes. As participants were Caucasian, these results may not be generalizable to other ethnicities. In future studies, it would be of interest to perform a time course to determine resolution of the miRNA levels in controls and whether miRNA changes showed a lag in T2D, occurring at a later time point. It is possible that the fundamental differences in the miRNA response between T2D and controls seen here could be due to a delayed response in T2D, an important point that should be addressed in future studies. Alternatively, it is important to bear in mind that hypoglycemia does not occur in normal controls and, in the face of this abnormal stimulus, then the response may be an abreaction. Of note, serum measurement of the individual miRNAs may not reflect cellular levels or activity. Assessment of the changes in miRNA at the tissue level are critical to determine the significance of the circulatory changes and their clinical application; whilst this was beyond the scope of our study, future studies should be undertaken to address this.

In conclusion, this study has shown, for the first time, rapid miRNA responses to hypoglycemia, of protective mechanisms in control subjects that appeared to be lost in T2D. Taken together, this suggests that mitigating responses to hypoglycemia with blunting of the counter-regulatory response in T2D occurs even in T2D with a short duration of disease.

## Data availability statement

The original contributions presented in the study are included in the article/**Supplementary Material**. Further inquiries can be directed to the corresponding author.

## Ethics statement

This study was reviewed and approved by the Yorkshire and the Humber Research Ethics Committee. The patients/participants provided their written informed consent to participate in this study.

## Author contributions

MR, RP, MB, JJ and KP performed miRNA measurements. TS performed clinical studies and edited the manuscript. AA-S supervised miRNA laboratory work. SA contributed to study design, data interpretation and the writing of the manuscript. NH performed the Ingenuity Pathway Analysis. AM and AB analyzed the data and wrote the manuscript. All authors reviewed and approved the final version of the manuscript. AB is the guarantor of this work. All authors contributed to the article and approved the submitted version.

## Conflict of interest

Authors MR, RP, MB,JJ and AA-S were employed by Hamad Medical Corporation

The remaining authors declare that the research was conducted in the absence of any commercial or financial relationships that could be construed as a potential conflict of interest.

## Publisher’s note

All claims expressed in this article are solely those of the authors and do not necessarily represent those of their affiliated organizations, or those of the publisher, the editors and the reviewers. Any product that may be evaluated in this article, or claim that may be made by its manufacturer, is not guaranteed or endorsed by the publisher.

## References

[1] Action to Control Cardiovascular Risk in Diabetes Study G Gerstein HC Miller ME Byington RP Goff DC Jr. Bigger JT. Effects of intensive glucose lowering in type 2 diabetes. N Engl J Med (2008) 358(24):2545–59. doi: 10.1056/NEJMoa0802743 PMC4551392 18539917

[2] Group AC Patel A MacMahon S Chalmers J Neal B Billot L. Intensive blood glucose control and vascular outcomes in patients with type 2 diabetes. N Engl J Med (2008) 358(24):2560–72. doi: 10.1056/NEJMoa0802987 18539916

[3] Miller ME Bonds DE Gerstein HC Seaquist ER Bergenstal RM Calles-Escandon J. The effects of baseline characteristics, glycaemia treatment approach, and glycated haemoglobin concentration on the risk of severe hypoglycaemia: post hoc epidemiological analysis of the ACCORD study. BMJ (2010) 340:b5444. doi: 10.1136/bmj.b5444 20061360 PMC2803743

[4] Bonds DE Miller ME Bergenstal RM Buse JB Byington RP Cutler JA. The association between symptomatic, severe hypoglycaemia and mortality in type 2 diabetes: retrospective epidemiological analysis of the ACCORD study. BMJ (2010) 340:b4909. doi: 10.1136/bmj.b4909 20061358 PMC2803744

[5] Investigators OT Mellbin LG Ryden L Riddle MC Probstfield J Rosenstock J. Does hypoglycaemia increase the risk of cardiovascular events? a report from the ORIGIN trial. Eur Heart J (2013) 34(40):3137–44. doi: 10.1093/eurheartj/eht332 23999452

[6] Investigators N-SS Finfer S Chittock DR Su SY Blair D Foster D. Intensive versus conventional glucose control in critically ill patients. N Engl J Med (2009) 360(13):1283–97. doi: 10.1056/NEJMoa0810625 19318384

[7] Bedenis R Price AH Robertson CM Morling JR Frier BM Strachan MW. Association between severe hypoglycemia, adverse macrovascular events, and inflammation in the Edinburgh type 2 diabetes study. Diabetes Care (2014) 37(12):3301–8. doi: 10.2337/dc14-0908 25239782

[8] Kosiborod M Inzucchi SE Goyal A Krumholz HM Masoudi FA Xiao L. Relationship between spontaneous and iatrogenic hypoglycemia and mortality in patients hospitalized with acute myocardial infarction. JAMA (2009) 301(15):1556–64. doi: 10.1001/jama.2009.496 19366775

[9] Goto A Arah OA Goto M Terauchi Y Noda M. Severe hypoglycaemia and cardiovascular disease: systematic review and meta-analysis with bias analysis. BMJ (2013) 347:f4533. doi: 10.1136/bmj.f4533 23900314

[10] Davis SN Duckworth W Emanuele N Hayward RA Wiitala WL Thottapurathu L. Effects of severe hypoglycemia on cardiovascular outcomes and death in the veterans affairs diabetes trial. Diabetes Care (2019) 42(1):157–63. doi: 10.2337/dc18-1144 PMC6463547 30455335

[11] Al-Qaissi A Papageorgiou M Deshmukh H Madden LA Rigby A Kilpatrick ES. Effects of acute insulin-induced hypoglycaemia on endothelial microparticles in adults with and without type 2 diabetes. Diabetes Obes Metab (2019) 21(3):533–40. doi: 10.1111/dom.13548 30264480

[12] Kahal H Aburima A Spurgeon B Wraith KS Rigby AS Sathyapalan T. Platelet function following induced hypoglycaemia in type 2 diabetes. Diabetes Metab (2018) 44:431–6. doi: 10.1016/j.diabet.2018.04.004 29784564

[13] Atkin AS Moin ASM Nandakumar M Al-Qaissi A Sathyapalan T Atkin SL. Impact of severe hypoglycemia on the heat shock and related protein response. Sci Rep (2021) 11(1):17057. doi: 10.1038/s41598-021-96642-8 34426634 PMC8382834

[14] Halama A Kahal H Bhagwat AM Zierer J Sathyapalan T Graumann J. Metabolic and proteomic signatures of hypoglycaemia in type 2 diabetes. Diabetes Obes Metab (2019) 21(4):909–19. doi: 10.1111/dom.13602 30525282

[15] Kahal H Halama A Aburima A Bhagwat AM Butler AE Graumann J. Effect of induced hypoglycemia on inflammation and oxidative stress in type 2 diabetes and control subjects. Sci Rep (2020) 10(1):4750. doi: 10.1038/s41598-020-61531-z 32179763 PMC7075968

[16] Atkin AS Moin ASM Al-Qaissi A Sathyapalan T Atkin SL Butler AE. Plasma heat shock protein response to euglycemia in type 2 diabetes. BMJ Open Diabetes Res Care (2021) 9(1):e002057. doi: 10.1136/bmjdrc-2020-002057 PMC8061861 33879515

[17] Lee Y Kim M Han J Yeom KH Lee S Baek SH. MicroRNA genes are transcribed by RNA polymerase II. EMBO J (2004) 23(20):4051–60. doi: 10.1038/sj.emboj.7600385 PMC524334 15372072

[18] Bartel DP. MicroRNAs: genomics, biogenesis, mechanism, and function. Cell (2004) 116(2):281–97. doi: 10.1016/S0092-8674(04)00045-5 14744438

[19] Kim VN Han J Siomi MC. Biogenesis of small RNAs in animals. Nat Rev Mol Cell Biol (2009) 10(2):126–39. doi: 10.1038/nrm2632 19165215

[20] Krol J Loedige I Filipowicz W. The widespread regulation of microRNA biogenesis, function and decay. Nat Rev Genet (2010) 11(9):597–610. doi: 10.1038/nrg2843 20661255

[21] Ortega FJ Mercader JM Catalan V Moreno-Navarrete JM Pueyo N Sabater M. Targeting the circulating microRNA signature of obesity. Clin Chem (2013) 59(5):781–92. doi: 10.1373/clinchem.2012.195776 23396142

[22] Atkin SL Ramachandran V Yousri NA Benurwar M Simper SC McKinlay R. Changes in blood microRNA expression and early metabolic responsiveness 21 days following bariatric surgery. Front Endocrinol (2018) 9:773. doi: 10.3389/fendo.2018.00773 PMC6338028 30687230

[23] Leung AK Sharp PA. MicroRNA functions in stress responses. Mol Cell (2010) 40(2):205–15. doi: 10.1016/j.molcel.2010.09.027 PMC2996264 20965416

[24] Witkowski M Weithauser A Tabaraie T Steffens D Kränkel N Witkowski M. Micro-RNA-126 reduces the blood thrombogenicity in diabetes mellitus *via* targeting of tissue factor. Arterioscler Thromb Vasc Biol (2016) 36(6):1263–71. doi: 10.1161/ATVBAHA.115.306094 PMC4894779 27127202

[25] Fulzele S El-Sherbini A Ahmad S Sangani R Matragoon S El-Remessy A. MicroRNA-146b-3p regulates retinal inflammation by suppressing adenosine deaminase-2 in diabetes. BioMed Res Int (2015) 2015:846501. doi: 10.1155/2015/846501 25815338 PMC4359882

[26] La Sala L Cattaneo M De Nigris V Pujadas G Testa R Bonfigli AR. Oscillating glucose induces microRNA-185 and impairs an efficient antioxidant response in human endothelial cells. Cardiovasc Diabetol (2016) 15:71. doi: 10.1186/s12933-016-0390-9 27137793 PMC4852407

[27] Caporali A Meloni M Völlenkle C Bonci D Sala-Newby GB Addis R. Deregulation of microRNA-503 contributes to diabetes mellitus-induced impairment of endothelial function and reparative angiogenesis after limb ischemia. Circulation (2011) 123(3):282–91. doi: 10.1161/CIRCULATIONAHA.110.952325 21220732

[28] Simone NL Soule BP Ly D Saleh AD Savage JE Degraff W. Ionizing radiation-induced oxidative stress alters miRNA expression. PLoS One (2009) 4(7):e6377. doi: 10.1371/journal.pone.0006377 19633716 PMC2712071

[29] Cui S Sun B Yin X Guo X Chao D Zhang C. Time-course responses of circulating microRNAs to three resistance training protocols in healthy young men. Sci Rep (2017) 7(1):2203. doi: 10.1038/s41598-017-02294-y 28526870 PMC5438360

[30] Favaro RR Morales-Prieto DM Herrmann J Sonnemann J Schleussner E Markert UR. Influence of high glucose in the expression of miRNAs and IGF1R signaling pathway in human myometrial explants. Arch Gynecol Obstet (2021) 303(6):1513–22. doi: 10.1007/s00404-020-05940-5 PMC8087607 33575847

[31] Zhang B-h Shen C-a Zhu B-w An H-y Zheng B Xu S-b. Insight into miRNAs related with glucometabolic disorder. Biomed Pharmacother (2019) 111:657–65. doi: 10.1016/j.biopha.2018.12.123 30611990

[32] Ueki S Murakami Y Yamada S Kimura M Saito Y Saito H. microRNA-mediated resistance to hypoglycemia in the HepG2 human hepatoma cell line. BMC Cancer (2016) 16(1):1–13. doi: 10.1186/s12885-016-2762-7 PMC5024426 27629773

[33] Mussa BM Taneera J Mohammed AK Srivastava A Mukhopadhyay D Sulaiman N. Potential role of hypothalamic microRNAs in regulation of FOS and FTO expression in response to hypoglycemia. J Physiol Sci (2019) 69(6):981–91. doi: 10.1007/s12576-019-00718-0 PMC10717546 31728912

[34] Agrawal R Durupt G Verma D Montgomery M Vieira-de Abreu A Taylor C. MicroRNA-7a overexpression in VMH restores the sympathoadrenal response to hypoglycemia. JCI Insight (2019) 4(20):e130521. doi: 10.1172/jci.insight.130521 PMC6824313 31619588

[35] Eyileten C Wicik Z Keshwani D Aziz F Aberer F Pferschy PN. Alteration of circulating platelet-related and diabetes-related microRNAs in individuals with type 2 diabetes mellitus: a stepwise hypoglycaemic clamp study. Cardiovasc Diabetol (2022) 21(1):1–12. doi: 10.1186/s12933-022-01517-5 35596173 PMC9123651

[36] Moin ASM Al-Qaissi A Sathyapalan T Atkin SL Butler AE. Hypoglycaemia in type 2 diabetes exacerbates amyloid-related proteins associated with dementia. Diabetes Obes Metab (2021) 23(2):338–49. doi: 10.1111/dom.14220 33026133

[37] Birkett MA Day SJ. Internal pilot studies for estimating sample size. Stat Med (1994) 13(23-24):2455–63. doi: 10.1002/sim.4780132309 7701146

[38] U.K. prospective diabetes study 16. overview of 6 years' therapy of type II diabetes: a progressive disease. U.K. prospective diabetes study group. Diabetes (1995) 44(11):1249–58. 7589820

[39] Sprague JE Arbeláez AM. Glucose counterregulatory responses to hypoglycemia. Pediatr Endocrinol Rev PER (2011) 9(1):463–75. PMC3755377 22783644

[40] Davis SN Mann S Briscoe VJ Ertl AC Tate DB. Effects of intensive therapy and antecedent hypoglycemia on counterregulatory responses to hypoglycemia in type 2 diabetes. Diabetes (2009) 58(3):701–9. doi: 10.2337/db08-1230 PMC2646069 19073776

[41] Chatterjee S Mudher A. Alzheimer's disease and type 2 diabetes: A critical assessment of the shared pathological traits. Front Neurosci (2018) 12:383. doi: 10.3389/fnins.2018.00383 29950970 PMC6008657

[42] Wang C Wan S Yang T Niu D Zhang A Yang C. Increased serum microRNAs are closely associated with the presence of microvascular complications in type 2 diabetes mellitus. Sci Rep (2016) 6:20032. doi: 10.1038/srep20032 26831044 PMC4735518

[43] Mayr B Müller EE Schäfer C Droese S Schönfelder M Niebauer J. Exercise-induced changes in miRNA expression in coronary artery disease. Clin Chem Lab Med CCLM / FESCC (2021) 59(10):1719–27. doi: 10.1515/cclm-2021-0164 33977686

[44] Kong L Du X Hu N Li W Wang W Wei S. Downregulation of let-7e-5p contributes to endothelial progenitor cell dysfunction in deep vein thrombosis *via* targeting FASLG. Thromb Res (2016) 138:30–6. doi: 10.1016/j.thromres.2015.12.020 26826505

[45] Müller-Deile J Dannenberg J Liu P Lorenzen J Nyström J Thum T. Identification of cell and disease specific microRNAs in glomerular pathologies. J Cell Mol Med (2019) 23(6):3927–39. doi: 10.1111/jcmm.14270 PMC6533525 30950172

[46] Pordzik J Jakubik D Jarosz-Popek J Wicik Z Eyileten C De Rosa S. Significance of circulating microRNAs in diabetes mellitus type 2 and platelet reactivity: bioinformatic analysis and review. Cardiovasc Diabetol (2019) 18(1):113. doi: 10.1186/s12933-019-0918-x 31470851 PMC6716825

[47] Cheng CC Yang BL Chen WC Ho AS Sie ZL Lin HC. STAT3 mediated miR-30a-5p inhibition enhances proliferation and inhibits apoptosis in colorectal cancer cells. Int J Mol Sci (2020) 21(19):7315. doi: 10.3390/ijms21197315 PMC7583989 33023006

[48] He Y Lang X Cheng D Zhang T Yang Z Xiong R. miR−30a−5p inhibits hypoxia/reoxygenation−induced oxidative stress and apoptosis in HK−2 renal tubular epithelial cells by targeting glutamate dehydrogenase 1 (GLUD1). Oncol Rep (2020) 44(4):1539–49. doi: 10.3892/or.2020.7718 PMC7448462 32945480

[49] Yang X Yang M Chen Y Qian Y Fei X Gong C. miR-30a-5p targets Becn1 to ameliorate high-glucose-induced glomerular podocyte injury in immortalized rat podocyte cell line. Am J Transl Res (2021) 13(3):1516–25. PMC8014343 33841675

[50] VatanIman R Malekpour SH Afshari A Zare M. MiR-770-5p, miR-661 and miR-571 expression level in serum and tissue samples of foot ulcer caused by diabetes mellitus type II in Iranian population. Mol Biol Rep (2021) 48(12):7811–8. doi: 10.1007/s11033-021-06798-9 34643918

[51] Wang L Li H. MiR-770-5p facilitates podocyte apoptosis and inflammation in diabetic nephropathy by targeting TIMP3. Biosci Rep (2020) 40(4):BSR20193653. doi: 10.1042/BSR20193653 32309847 PMC7189364

[52] Zhang J Song L Ma Y Yin Y Liu X Luo X. lncRNA MEG8 upregulates miR-770-5p through methylation and promotes cell apoptosis in diabetic nephropathy. Diabetes Metab Syndr Obes: Targets Ther (2020) 13:2477. doi: 10.2147/DMSO.S255183 PMC7360416 32765026

[53] Jiang L Yu L Zhang X Lei F Wang L Liu X. miR-892b silencing activates NF-κB and promotes aggressiveness in breast cancer. Cancer Res (2016) 76(5):1101–11. doi: 10.1158/0008-5472.CAN-15-1770 26747895

[54] Villard A Marchand L Thivolet C Rome S. Diagnostic value of cell-free circulating microRNAs for obesity and type 2 diabetes: a meta-analysis. J Mol Biomarkers Diagn (2015) 6(6):251. doi: 10.4172/2155-9929.1000251 PMC4905583 27308097

[55] Liang L Su W Zhou L Cao Y Zhou X Liu S. Statin downregulation of miR-652-3p protects endothelium from dyslipidemia by promoting ISL1 expression. Metabol: Clin Exp (2020) 107:154226. doi: 10.1016/j.metabol.2020.154226 32277945

