# Supplementary material for: MiRNA and associated inflammatory changes from baseline to hypoglycemia in type 2 diabetes
Source: Front Endocrinol (Lausanne). 2022 Aug 9;13:917041. doi: 10.3389/fendo.2022.917041 (PMC9395634; doi:10.3389/fendo.2022.917041)
Supplement: Supplementary file 1 [file Table_1.docx]

**Supplementary table 1**. Baseline Rq values for the 96 miRNAs in the validation experiment. The data demonstrates that there were no differences in miRNAs between type 2 diabetes (T2D) and control subjects at baseline. Control Rq was used as a reference and hence its value for each miRNA equals 1. BL, baseline; Rq, relative level of miRNA expression.

| Target Name | Control_BL (Rq) | T2D_BL (Rq) | P-Value |
| --- | --- | --- | --- |
| hsa-let-7b-5p_478576_mir | 1 | 0.97 | 0.87 |
| hsa-let-7d-3p_477848_mir | 1 | 0.47 | 0.25 |
| hsa-let-7e-5p_478579_mir | 1 | 1.35 | 0.44 |
| hsa-let-7g-5p_478580_mir | 1 | 0.88 | 0.35 |
| hsa-miR-101-3p_477863_mir | 1 | 0.84 | 0.51 |
| hsa-miR-106b-3p_477866_mir | 1 | 0.78 | 0.37 |
| hsa-miR-106b-5p_478412_mir | 1 | 0.53 | 0.50 |
| hsa-miR-10a-5p_479241_mir | 1 | 1.20 | 0.52 |
| hsa-miR-125a-5p_477884_mir | 1 | 1.14 | 0.52 |
| hsa-miR-125b-5p_477885_mir | 1 | 1.02 | 0.93 |
| hsa-miR-126-3p_477887_mir | 1 | 0.96 | 0.92 |
| hsa-miR-126-5p_477888_mir | 1 | 0.96 | 0.79 |
| hsa-miR-1267_478672_mir | 1 | 1.35 | 0.44 |
| hsa-miR-128-3p_477892_mir | 1 | 0.99 | 0.94 |
| hsa-miR-1303_478698_mir | 1 | 1.70 | 0.12 |
| hsa-miR-130a-3p_477851_mir | 1 | 0.93 | 0.58 |
| hsa-miR-130b-3p_477840_mir | 1 | 0.72 | 0.47 |
| hsa-miR-140-3p_477908_mir | 1 | 1.07 | 0.76 |
| hsa-miR-143-3p_477912_mir | 1 | 0.87 | 0.49 |
| hsa-miR-144-3p_477913_mir | 1 | 0.73 | 0.30 |
| hsa-miR-146a-5p_478399_mir | 1 | 1.09 | 0.69 |
| hsa-miR-148a-3p_477814_mir | 1 | 1.09 | 0.55 |
| hsa-miR-148b-3p_477824_mir | 1 | 0.97 | 0.82 |
| hsa-miR-150-5p_477918_mir | 1 | 1.10 | 0.72 |
| hsa-miR-151a-3p_477919_mir | 1 | 0.81 | 0.30 |
| hsa-miR-152-3p_477921_mir | 1 | 0.77 | 0.57 |
| hsa-miR-15a-5p_477858_mir | 1 | 0.74 | 0.17 |
| hsa-miR-15b-3p_477929_mir | 1 | 0.29 | 0.08 |
| hsa-miR-16-2-3p_477931_mir | 1 | 0.77 | 0.40 |
| hsa-miR-16-5p_477860_mir | 1 | 0.73 | 0.25 |
| hsa-miR-17-5p_478447_mir | 1 | 0.73 | 0.14 |
| hsa-miR-181a-5p_477857_mir | 1 | 0.90 | 0.61 |
| hsa-miR-181b-5p_478583_mir | 1 | 0.90 | 0.61 |
| hsa-miR-185-5p_477939_mir | 1 | 0.82 | 0.36 |
| hsa-miR-186-5p_477940_mir | 1 | 0.85 | 0.24 |
| hsa-miR-191-5p_477952_mir | 1 | 0.84 | 0.26 |
| hsa-miR-194-5p_477956_mir | 1 | 1.47 | 0.06 |
| hsa-miR-195-5p_477957_mir | 1 | 1.18 | 0.41 |
| hsa-miR-199a-3p_477961_mir | 1 | 0.96 | 0.84 |
| hsa-miR-20a-5p_478586_mir | 1 | 0.72 | 0.07 |
| hsa-miR-21-5p_477975_mir | 1 | 0.865 | 0.212 |
| hsa-miR-210-3p_477970_mir | 1 | 0.976 | 0.925 |
| hsa-miR-215-5p_478516_mir | 1 | 0.67 | 0.504 |
| hsa-miR-22-3p_477985_mir | 1 | 0.914 | 0.614 |
| hsa-miR-22-5p_477987_mir | 1 | 0.979 | 0.875 |
| hsa-miR-221-3p_477981_mir | 1 | 0.886 | 0.482 |
| hsa-miR-222-3p_477982_mir | 1 | 0.846 | 0.153 |
| hsa-miR-223-3p_477983_mir | 1 | 0.951 | 0.822 |
| hsa-miR-24-3p_477992_mir | 1 | 1.029 | 0.875 |
| hsa-miR-25-3p_477994_mir | 1 | 0.705 | 0.166 |
| hsa-miR-26a-5p_477995_mir | 1 | 0.542 | 0.253 |
| hsa-miR-27a-3p_478384_mir | 1 | 0.988 | 0.956 |
| hsa-miR-27b-3p_478270_mir | 1 | 0.858 | 0.508 |
| hsa-miR-29b-3p_478369_mir | 1 | 0.961 | 0.662 |
| hsa-miR-30a-5p_479448_mir | 1 | 1.35 | 0.441 |
| hsa-miR-324-5p_478024_mir | 1 | 0.828 | 0.248 |
| hsa-miR-328-3p_478028_mir | 1 | 1.258 | 0.133 |
| hsa-miR-338-3p_478037_mir | 1 | 1.178 | 0.496 |
| hsa-miR-339-5p_478040_mir | 1 | 0.667 | 0.341 |
| hsa-miR-342-3p_478043_mir | 1 | 1.08 | 0.696 |
| hsa-miR-361-5p_478056_mir | 1 | 0.994 | 0.973 |
| hsa-miR-363-3p_478060_mir | 1 | 1.019 | 0.951 |
| hsa-miR-365a-3p_478065_mir | 1 | 1.421 | 0.695 |
| hsa-miR-369-3p_478067_mir | 1 | 0.824 | 0.689 |
| hsa-miR-375-3p_478074_mir | 1 | 1.223 | 0.825 |
| hsa-miR-378a-3p_478349_mir | 1 | 0.92 | 0.839 |
| hsa-miR-378a-5p_478076_mir | 1 | 0.853 | 0.767 |
| hsa-miR-409-3p_478084_mir | 1 | 1.246 | 0.595 |
| hsa-miR-410-3p_478085_mir | 1 | 0.852 | 0.825 |
| hsa-miR-423-5p_478090_mir | 1 | 0.937 | 0.654 |
| hsa-miR-424-5p_478092_mir | 1 | 1.277 | 0.061 |
| hsa-miR-425-3p_478093_mir | 1 | 1.171 | 0.695 |
| hsa-miR-425-5p_478094_mir | 1 | 0.809 | 0.281 |
| hsa-miR-431-5p_478889_mir | 1 | 0.811 | 0.79 |
| hsa-miR-483-3p_478122_mir | 1 | 1.109 | 0.91 |
| hsa-miR-484_478308_mir | 1 | 0.909 | 0.438 |
| hsa-miR-495-3p_478136_mir | 1 | 1.069 | 0.826 |
| hsa-miR-505-3p_478145_mir | 1 | 1.282 | 0.189 |
| hsa-miR-517a-3p_479485_mir | 1 | 1.512 | 0.399 |
| hsa-miR-571_479054_mir | 1 | 1.35 | 0.441 |
| hsa-miR-576-5p_478165_mir | 1 | 0.787 | 0.524 |
| hsa-miR-584-5p_478167_mir | 1 | 0.927 | 0.887 |
| hsa-miR-590-5p_478367_mir | 1 | 0.454 | 0.146 |
| hsa-miR-652-3p_478189_mir | 1 | 0.839 | 0.202 |
| hsa-miR-660-5p_478192_mir | 1 | 0.724 | 0.298 |
| hsa-miR-661_479144_mir | 1 | 1.35 | 0.441 |
| hsa-miR-7-5p_478341_mir | 1 | 0.909 | 0.898 |
| hsa-miR-770-5p_479178_mir | 1 | 1.35 | 0.441 |
| hsa-miR-885-5p_478207_mir | 1 | 1.268 | 0.665 |
| hsa-miR-892b_479198_mir | 1 | 1.35 | 0.441 |
| hsa-miR-92a-3p_477827_mir | 1 | 0.79 | 0.24 |
| hsa-miR-92b-3p_477823_mir | 1 | 0.943 | 0.768 |
| hsa-miR-93-5p_478210_mir | 1 | 0.787 | 0.251 |
| hsa-miR-99b-5p_478343_mir | 1 | 1.587 | 0.38 |
