# Supplementary material for: MiRNA and associated inflammatory changes from baseline to hypoglycemia in type 2 diabetes
Source: Front Endocrinol (Lausanne). 2022 Aug 9;13:917041. doi: 10.3389/fendo.2022.917041 (PMC9395634; doi:10.3389/fendo.2022.917041)
Supplement: Supplementary file 2 [file Table_2_v1.docx]

**Supplementary table 2.** Changes in all miRs from baseline to hypoglycemia in T2D and controls in the validation experiment. Rq, relative level of miRNA expression.

| **CONTROLS** |  |  |  | **T2D** |  |  |
| --- | --- | --- | --- | --- | --- | --- |
| **Target Name** | **Rq** | **P-Value** |  | **Target Name** | **Rq** | **P-Value** |
| hsa-miR-1303_478698_mir | 2.264 | 0.013 |  | hsa-miR-106b-5p_478412_mir | 4.598 | 0.064 |
| hsa-miR-652-3p_478189_mir | 0.767 | 0.017 |  | hsa-miR-194-5p_477956_mir | 0.702 | 0.11 |
| hsa-let-7e-5p_478579_mir | 1.934 | 0.028 |  | hsa-let-7g-5p_478580_mir | 1.185 | 0.144 |
| hsa-miR-1267_478672_mir | 1.934 | 0.028 |  | hsa-let-7d-3p_477848_mir | 2.609 | 0.154 |
| hsa-miR-30a-5p_479448_mir | 1.934 | 0.028 |  | hsa-miR-181b-5p_478583_mir | 1.317 | 0.166 |
| hsa-miR-571_479054_mir | 1.934 | 0.028 |  | hsa-miR-152-3p_477921_mir | 1.878 | 0.167 |
| hsa-miR-661_479144_mir | 1.934 | 0.028 |  | hsa-miR-195-5p_477957_mir | 0.789 | 0.172 |
| hsa-miR-770-5p_479178_mir | 1.934 | 0.028 |  | hsa-miR-181a-5p_477857_mir | 1.36 | 0.178 |
| hsa-miR-892b_479198_mir | 1.934 | 0.028 |  | hsa-miR-222-3p_477982_mir | 1.141 | 0.193 |
| hsa-miR-423-5p_478090_mir | 0.807 | 0.065 |  | hsa-miR-342-3p_478043_mir | 1.322 | 0.208 |
| hsa-miR-484_478308_mir | 0.854 | 0.084 |  | hsa-miR-99b-5p_478343_mir | 1.606 | 0.221 |
| hsa-miR-92a-3p_477827_mir | 0.746 | 0.088 |  | hsa-miR-378a-5p_478076_mir | 2.113 | 0.24 |
| hsa-miR-517a-3p_479485_mir | 1.588 | 0.137 |  | hsa-miR-130b-3p_477840_mir | 1.736 | 0.242 |
| hsa-miR-25-3p_477994_mir | 0.737 | 0.151 |  | hsa-miR-126-3p_477887_mir | 1.202 | 0.253 |
| hsa-miR-143-3p_477912_mir | 1.222 | 0.18 |  | hsa-miR-26a-5p_477995_mir | 1.832 | 0.263 |
| hsa-miR-22-5p_477987_mir | 1.129 | 0.18 |  | hsa-miR-186-5p_477940_mir | 1.185 | 0.266 |
| hsa-miR-151a-3p_477919_mir | 0.83 | 0.202 |  | hsa-miR-150-5p_477918_mir | 1.374 | 0.274 |
| hsa-let-7b-5p_478576_mir | 0.809 | 0.222 |  | hsa-miR-369-3p_478067_mir | 1.677 | 0.289 |
| hsa-miR-215-5p_478516_mir | 1.481 | 0.241 |  | hsa-miR-378a-3p_478349_mir | 1.398 | 0.294 |
| hsa-miR-186-5p_477940_mir | 0.872 | 0.249 |  | hsa-miR-140-3p_477908_mir | 0.855 | 0.304 |
| hsa-miR-338-3p_478037_mir | 1.256 | 0.258 |  | hsa-miR-128-3p_477892_mir | 1.168 | 0.345 |
| hsa-miR-10a-5p_479241_mir | 1.283 | 0.295 |  | hsa-miR-210-3p_477970_mir | 0.693 | 0.357 |
| hsa-miR-424-5p_478092_mir | 1.115 | 0.338 |  | hsa-miR-483-3p_478122_mir | 2.205 | 0.369 |
| hsa-miR-17-5p_478447_mir | 0.848 | 0.356 |  | hsa-miR-339-5p_478040_mir | 1.47 | 0.374 |
| hsa-miR-15b-3p_477929_mir | 0.802 | 0.363 |  | hsa-miR-148a-3p_477814_mir | 0.872 | 0.382 |
| hsa-miR-92b-3p_477823_mir | 0.852 | 0.373 |  | hsa-miR-15a-5p_477858_mir | 1.212 | 0.397 |
| hsa-miR-152-3p_477921_mir | 1.251 | 0.382 |  | hsa-miR-361-5p_478056_mir | 1.201 | 0.397 |
| hsa-miR-27a-3p_478384_mir | 1.149 | 0.404 |  | hsa-miR-1303_478698_mir | 0.732 | 0.404 |
| hsa-miR-16-2-3p_477931_mir | 0.814 | 0.417 |  | hsa-miR-584-5p_478167_mir | 1.54 | 0.425 |
| hsa-miR-324-5p_478024_mir | 0.901 | 0.417 |  | hsa-miR-151a-3p_477919_mir | 1.189 | 0.44 |
| hsa-miR-140-3p_477908_mir | 1.178 | 0.431 |  | hsa-miR-424-5p_478092_mir | 0.908 | 0.441 |
| hsa-miR-223-3p_477983_mir | 1.135 | 0.485 |  | hsa-miR-576-5p_478165_mir | 0.699 | 0.461 |
| hsa-miR-425-3p_478093_mir | 1.229 | 0.497 |  | hsa-miR-221-3p_477981_mir | 1.166 | 0.462 |
| hsa-miR-191-5p_477952_mir | 0.918 | 0.499 |  | hsa-miR-885-5p_478207_mir | 1.441 | 0.503 |
| hsa-miR-378a-5p_478076_mir | 0.752 | 0.502 |  | hsa-miR-590-5p_478367_mir | 1.529 | 0.506 |
| hsa-miR-148a-3p_477814_mir | 1.085 | 0.516 |  | hsa-miR-22-3p_477985_mir | 1.102 | 0.523 |
| hsa-miR-20a-5p_478586_mir | 0.903 | 0.523 |  | hsa-miR-21-5p_477975_mir | 1.077 | 0.536 |
| hsa-miR-24-3p_477992_mir | 1.091 | 0.527 |  | hsa-miR-652-3p_478189_mir | 1.113 | 0.537 |
| hsa-miR-505-3p_478145_mir | 1.127 | 0.53 |  | hsa-miR-7-5p_478341_mir | 0.582 | 0.54 |
| hsa-miR-99b-5p_478343_mir | 1.395 | 0.531 |  | hsa-miR-328-3p_478028_mir | 0.899 | 0.542 |
| hsa-miR-16-5p_477860_mir | 0.869 | 0.547 |  | hsa-miR-505-3p_478145_mir | 1.188 | 0.549 |
| hsa-miR-130b-3p_477840_mir | 1.127 | 0.56 |  | hsa-let-7e-5p_478579_mir | 1.421 | 0.551 |
| hsa-miR-425-5p_478094_mir | 0.91 | 0.567 |  | hsa-miR-338-3p_478037_mir | 0.824 | 0.553 |
| hsa-miR-194-5p_477956_mir | 1.12 | 0.572 |  | hsa-miR-126-5p_477888_mir | 1.111 | 0.556 |
| hsa-miR-150-5p_477918_mir | 1.133 | 0.575 |  | hsa-miR-27b-3p_478270_mir | 1.168 | 0.57 |
| hsa-miR-106b-5p_478412_mir | 1.545 | 0.581 |  | hsa-miR-660-5p_478192_mir | 1.202 | 0.587 |
| hsa-miR-93-5p_478210_mir | 0.916 | 0.598 |  | hsa-miR-223-3p_477983_mir | 1.139 | 0.596 |
| hsa-miR-222-3p_477982_mir | 0.939 | 0.606 |  | hsa-miR-125a-5p_477884_mir | 1.12 | 0.61 |
| hsa-miR-126-5p_477888_mir | 0.944 | 0.616 |  | hsa-miR-15b-3p_477929_mir | 1.539 | 0.623 |
| hsa-miR-660-5p_478192_mir | 0.89 | 0.628 |  | hsa-miR-199a-3p_477961_mir | 1.134 | 0.634 |
| hsa-let-7d-3p_477848_mir | 0.874 | 0.643 |  | hsa-miR-410-3p_478085_mir | 1.484 | 0.639 |
| hsa-miR-369-3p_478067_mir | 0.841 | 0.646 |  | hsa-miR-106b-3p_477866_mir | 1.122 | 0.64 |
| hsa-miR-15a-5p_477858_mir | 0.923 | 0.66 |  | hsa-miR-484_478308_mir | 1.065 | 0.649 |
| hsa-miR-181b-5p_478583_mir | 1.069 | 0.663 |  | hsa-miR-191-5p_477952_mir | 1.089 | 0.652 |
| hsa-miR-483-3p_478122_mir | 1.386 | 0.664 |  | hsa-miR-148b-3p_477824_mir | 0.918 | 0.656 |
| hsa-miR-885-5p_478207_mir | 1.156 | 0.674 |  | hsa-miR-22-5p_477987_mir | 1.066 | 0.665 |
| hsa-miR-128-3p_477892_mir | 0.956 | 0.685 |  | hsa-miR-146a-5p_478399_mir | 1.113 | 0.666 |
| hsa-miR-125a-5p_477884_mir | 1.071 | 0.69 |  | hsa-let-7b-5p_478576_mir | 0.913 | 0.668 |
| hsa-miR-125b-5p_477885_mir | 1.051 | 0.706 |  | hsa-miR-365a-3p_478065_mir | 1.462 | 0.679 |
| hsa-miR-29b-3p_478369_mir | 1.034 | 0.709 |  | hsa-miR-363-3p_478060_mir | 0.912 | 0.688 |
| hsa-miR-27b-3p_478270_mir | 1.061 | 0.722 |  | hsa-miR-130a-3p_477851_mir | 0.935 | 0.7 |
| hsa-miR-181a-5p_477857_mir | 1.055 | 0.739 |  | hsa-miR-125b-5p_477885_mir | 1.084 | 0.727 |
| hsa-miR-342-3p_478043_mir | 1.062 | 0.745 |  | hsa-miR-10a-5p_479241_mir | 1.09 | 0.734 |
| hsa-miR-185-5p_477939_mir | 0.942 | 0.746 |  | hsa-miR-24-3p_477992_mir | 1.07 | 0.741 |
| hsa-miR-339-5p_478040_mir | 1.044 | 0.746 |  | hsa-miR-425-3p_478093_mir | 0.86 | 0.742 |
| hsa-miR-584-5p_478167_mir | 0.913 | 0.751 |  | hsa-miR-375-3p_478074_mir | 0.714 | 0.751 |
| hsa-miR-126-3p_477887_mir | 1.035 | 0.758 |  | hsa-miR-27a-3p_478384_mir | 1.076 | 0.779 |
| hsa-miR-410-3p_478085_mir | 1.188 | 0.774 |  | hsa-miR-25-3p_477994_mir | 1.077 | 0.784 |
| hsa-miR-431-5p_478889_mir | 0.816 | 0.774 |  | hsa-miR-101-3p_477863_mir | 0.939 | 0.791 |
| hsa-miR-148b-3p_477824_mir | 0.965 | 0.777 |  | hsa-miR-517a-3p_479485_mir | 0.872 | 0.813 |
| hsa-let-7g-5p_478580_mir | 0.964 | 0.786 |  | hsa-miR-144-3p_477913_mir | 0.939 | 0.816 |
| hsa-miR-7-5p_478341_mir | 0.855 | 0.792 |  | hsa-miR-409-3p_478084_mir | 1.089 | 0.829 |
| hsa-miR-363-3p_478060_mir | 1.072 | 0.797 |  | hsa-miR-185-5p_477939_mir | 1.045 | 0.835 |
| hsa-miR-365a-3p_478065_mir | 1.166 | 0.827 |  | hsa-miR-1267_478672_mir | 0.92 | 0.841 |
| hsa-miR-22-3p_477985_mir | 1.033 | 0.833 |  | hsa-miR-30a-5p_479448_mir | 0.92 | 0.841 |
| hsa-miR-590-5p_478367_mir | 0.966 | 0.877 |  | hsa-miR-571_479054_mir | 0.92 | 0.841 |
| hsa-miR-495-3p_478136_mir | 0.952 | 0.877 |  | hsa-miR-661_479144_mir | 0.92 | 0.841 |
| hsa-miR-375-3p_478074_mir | 0.907 | 0.888 |  | hsa-miR-770-5p_479178_mir | 0.92 | 0.841 |
| hsa-miR-576-5p_478165_mir | 0.963 | 0.891 |  | hsa-miR-892b_479198_mir | 0.92 | 0.841 |
| hsa-miR-210-3p_477970_mir | 0.974 | 0.9 |  | hsa-miR-16-5p_477860_mir | 0.96 | 0.871 |
| hsa-miR-101-3p_477863_mir | 1.026 | 0.911 |  | hsa-miR-324-5p_478024_mir | 1.028 | 0.883 |
| hsa-miR-106b-3p_477866_mir | 1.026 | 0.912 |  | hsa-miR-143-3p_477912_mir | 0.966 | 0.884 |
| hsa-miR-378a-3p_478349_mir | 0.953 | 0.913 |  | hsa-miR-20a-5p_478586_mir | 1.025 | 0.887 |
| hsa-miR-199a-3p_477961_mir | 1.015 | 0.941 |  | hsa-miR-423-5p_478090_mir | 1.025 | 0.889 |
| hsa-miR-195-5p_477957_mir | 1.011 | 0.949 |  | hsa-miR-17-5p_478447_mir | 1.03 | 0.892 |
| hsa-miR-409-3p_478084_mir | 0.975 | 0.949 |  | hsa-miR-93-5p_478210_mir | 0.975 | 0.9 |
| hsa-miR-26a-5p_477995_mir | 0.993 | 0.95 |  | hsa-miR-92b-3p_477823_mir | 0.974 | 0.914 |
| hsa-miR-146a-5p_478399_mir | 1.009 | 0.963 |  | hsa-miR-92a-3p_477827_mir | 1.016 | 0.945 |
| hsa-miR-221-3p_477981_mir | 1.006 | 0.966 |  | hsa-miR-431-5p_478889_mir | 1.062 | 0.951 |
| hsa-miR-328-3p_478028_mir | 0.995 | 0.967 |  | hsa-miR-29b-3p_478369_mir | 1.007 | 0.953 |
| hsa-miR-144-3p_477913_mir | 0.991 | 0.974 |  | hsa-miR-16-2-3p_477931_mir | 1.018 | 0.956 |
| hsa-miR-361-5p_478056_mir | 1.002 | 0.988 |  | hsa-miR-425-5p_478094_mir | 1.007 | 0.967 |
| hsa-miR-21-5p_477975_mir | 1.001 | 0.993 |  | hsa-miR-215-5p_478516_mir | 0.971 | 0.969 |
| hsa-miR-130a-3p_477851_mir | 1 | 0.999 |  | hsa-miR-495-3p_478136_mir | 1.003 | 0.993 |
